# Comparison of Ondansetron versus Clonidine efficacy for prevention of postoperative pain, nausea and vomiting after orthognathic surgeries: A triple blind randomized controlled trial

**DOI:** 10.4317/medoral.22493

**Published:** 2018-11-21

**Authors:** Sahand Samieirad, Alireza Sharifian-Attar, Majid Eshghpour, Vajiheh Mianbandi, Elaheh Shadkam, Majid Hosseini-Abrishami, Mayam-Sadat Hashemipour

**Affiliations:** 1 Assistant Professor, Oral and maxillofacial diseases research center, Mashhad University of Medical Sciences, Mashhad, Iran; 2 Assistant Professor, Oral and maxillofacial surgery department, Mashhad dental school, Mashhad University of medical sciences, Mashhad, Iran; 3 Associate Professor, Anesthesiology department, Medical faculty, Mashhad University of Medical Sciences, Mashhad, Iran; 4 Associate Professor, Oral and maxillofacial surgery department, Mashhad dental school, Mashhad University of medical sciences, Mashhad, Iran; 5 Dentistry Student, Student Research Committee, Mashhad dental school, Mashhad University of Medical Sciences, Mashhad, Iran; 6 Post-graduate Student in orthodontics, Student Research Committee, Mashhad dental school, Mashhad University of Medical Sciences, Mashhad, Iran; 7 DDS, MSc. Member of Kerman Dental and Oral Diseases Research Center, Kerman University of Medical Sciences, Kerman, Iran. Associate Professor of Oral Medicine, Department of Oral Medicine, Dental School, Kerman University of Medical Science, Kerman, Iran

## Abstract

**Background:**

The aim of this randomized controlled triple blind trial was to compare the efficacy of clonidine with dexamethasone versus ondansetron with dexamethasone for postoperative pain, nausea and vomiting prevention in orthognathic surgery patients.

**Material and Methods:**

In this clinical trial study, 30 consecutive patients with skeletal class III deformities were candidates for orthognathic surgery in Qaem hospital, Mashhad University of medical sciences, Mashhad, Iran from March to November 2017. These subjects were randomly assigned to two equal number groups, ondansetron or clonidine. Patients received either oral ondansetron 8mg or oral clonidine 150μg as premedication, 1 hour before the surgery (both dissolved in 20 cc of water). Also both groups received intravenous dexamethasone 8mg (1 hour preoperatively and every 4 hours intraoperatively).

**Results:**

In this study, a total of 30 patients (14 males and 16 females) with a mean age of 23.9 ± 3.9 were investigated. The incidence of postoperative nausea in women was more than men (*p*=0.003), also the correlation between the incidence of PON and the surgery duration ≥ 3 hours was statistically significant (*p* = 0.050). The frequency of postoperative nausea (PON) in the ondansetron group was less than clonidine (53.3% vs 73.3% respectively). There was no postoperative vomiting (POV) in the ondansetron group, but 6.7% of cases in clonidine group suffered POV. Post-operative nausea in ondansetron group occurred significantly later than clonidine (525.0±233.2 vs 100.0±34.0 min; *p*<0.001). On the other hand, the incidence time of post-operative severe pain or in other word the analgesia time in clonidine group was significantly more than ondansetron one (875/0±68/5 vs 614.3±159.1 min; *p*<0.001).

**Conclusions:**

Ondansetron with dexamethasone premedication was more effective in controlling PONV after orthognathic surgery compared to clonidine with dexamethasone group.

** Key words:**Postoperative nausea and vomiting, ondansetron, clonidine.

## Introduction

Postoperative Nausea and Vomiting (PONV) is considered as a distressing and challenging complication of post-surgery and anesthesia (1-7). It is of greater concern than even postoperative pain, to the surgical patients(1-3). PONV may lead to serious surgical complications such as wound dehiscence or surgical site bleeding, hematoma, aspiration and choking probability, water and electrolyte disturbances resulting in increased healthcare costs due to lower patient satisfaction and delayed hospital discharge (1-5,8,9).

It is believed that PONV has multifactorial origin (1-3,5,7,8). These factors can be divided into patient-related characteristics (age, gender, obesity and smoking status as well as history of motion sickness or previous PONV), anesthetic-related (volatile anesthetics, intraoperative use of opioid and blood transfusion) and surgical-related factors (type and duration of surgery and use of postoperative analgesic opioids) (1-3,5,7,8).

The estimated incidence of PONV after general surgeries would be approximately 40-60%, which can reach up to %80 in high risk patients underlying the importance of its prevention and control by surgeons and anesthetists. Regarding the recent articles, the estimated PONV incidence during the first 24 h after orthognathic surgeries is stated to be 40-68% (1-3,9).

PONV prevention for orthognathic surgical patients remains an under-investigated domain of clinical care to maximize patient safety and satisfaction (1,2). Maxillomandibular elastic tractions and intermaxillary wires following orthognathic surgery can magnify the anxiety, agitation and risk of aspiration associated with PONV, which may be life threatening event in maxillofacial patients (1,10).

As a matter of fact, administration of antiemetic drugs to prevent and manage PONV is an important tool that should be properly used (1,2,4,5,8,11). Considering the multifactorial etiology of this complication, multimodal therapy is recommended for prevention of PONV in high risk patients. Therefore various drug combinations have been tried and found to be effective for this issue, albeit they had some side effects (1,2,5,12).

Clonidine is a α2-adrenergic agonist drug, playing an important role as a sedative in anesthesia and pain control which would act more effectively than opioids and benzodiazepines in decreasing central and peripheral blood pressure with lower adverse effects (5,6,13). It has been reported to prevent the sympatho-adrenergic response to anesthesia, suppress cardiovascular response to laryngoscopy and reduce anesthetic and analgesic requirements (13,14). It also provides preoperative sedation, postoperative analgesia and perioperative hemodynamic stability (5,6,10,11,13,14).

In addition, recent investigations have demonstrated that premedication with oral clonidine is successful in reducing the PONV after strabismus, ear and breast cancer surgeries as well as appendectomy and thyroid surgeries due to its multifactorial influence on decreasing stress, general sympathetic tone and catecholamine release (5-7,10,11,13).

Ondansetron is an antagonist for serotonin (5HT3: 5-hydroxy tryptamine subtype 3) receptor. It is well established for prophylaxis of PONV as oral and intravenous preparation (5,15). Due to its strong anti-vomiting effect without complications such as bradycardia and extrapyramidal syndrome, ondansetron is applied in several studies to reduce PONV after chemotherapy, laparoscopy and strabismus surgeries as well as ENT and thyroid surgeries (4,5,8,16,17). Interestingly, the recent reports declared ondansetron might attach to µ opioid receptor as an antagonist that leads to analgesic effects especially in neuropathic pains (15,18-20).

It is noteworthy that intravenous Dexamethasone has been combined with other drugs for successful prophylaxis of PONV, according to multimodal protocol in recent investigations (4,5,8,12).

The epidemiology of PONV is well described in the literature with regard to abdominal, gynecological, plastic, ENT and other types of general surgeries (4,6-8,11,12). However only limited studies specifically analyzed the prevalence of PONV, related risk factors and its management among orthognathic surgery patients (1-3). PONV prevention for orthognathic surgical patients remains an under-investigated domain of clinical care (1,2).

To the best of our knowledge, no article comparing the efficacy of Ondansetron versus Clonidine on post-operative pain, vomiting and nausea management after orthognathic surgeries has been published up to now, except Shilpa’s study after thyroidectomy (5).

Hence, we decided to perform this randomized triple blind clinical study to investigate and compare the efficacy of oral ondansetron with intravenous dexamethasone versus oral clonidine with intravenous dexamethasone for prevention of postoperative pain and PONV after orthognathic surgeries.

## Material and Methods

A triple-blind randomized clinical trial was carried out. The protocol of this study was approved by the Ethics and Research Committee of Mashhad University of Medical Sciences (IR.mums.sd.REC.1394.176), and was registered in IRCT under the code IRCT2017012922697N2. Guidelines of the Declaration of Helsinki were followed in this research.

After obtaining written informed consent, all the healthy systemic patients (ASA I, II) who had the skeletal class III deformities with the age range of 18 to 45 and BMI less than 30 kg/ (non-obese) from any gender or race were included in this study. These cases were candidates for orthognathic surgery in maxillofacial surgery department of Qaem Hospital of Mashhad (Iran), from March to November 2017.

Subjects with serious medical conditions (ASA≥III), history of previous motion sickness or recent emesis, hypersensitivity and allergy to any study drugs were excluded from this study. In addition, the patients showing severe hypotension and bradycardia during the surgery and requiring blood transfusion intra-operatively or ICU post-operatively as well as those who refused the follow-up checkups were excluded too. None of the patients were smokers.

All of the operations were carried out by a single surgeon and anesthesiologist using the same protocol. A pre-anesthetic and pre-operative evaluations were done in all patients with a detailed history, routine laboratory and radiographic investigations. Also the type of deformities and surgical treatment plans as well as Patients’ ages, sexes and BMIs were determined and recorded in checklist.

The cases were divided into two equal number groups (ondansetron or clonidine) by balanced block randomization. In line with Consort guidelines, random codes were applied according to the number of patients and drugs, so each patient was randomly categorized with a code. Allocation concealment was performed using sequentially numbered opaque sealed envelopes.

The first group was given 150μg clonidine tablet dissolved in 20 cc water orally 60 min before surgery. The second group was given 8mg ondansetron tablet similar to the first group dissolved in 20cc water at the same time.

In addition, 8 mg intravenous dexamethasone was injected for both groups, 60 min preoperatively.

All the packages were labeled and numbered randomly. Assigning the patients to either of the treatment groups randomlyand administration of the drugs was done by the student who was not involved in the surgery and anesthesia procedure.

Prior to the surgery each patient was given a package and the aim of the study was explained to the patients at the start, but they were not told who would receive which drug. The randomization code was concealed from the surgeon, study investigator (nursing staff) and patients. The codes kept in a secure location until the end of the study.

In other word, neither the patient nor the surgeon and study investigator were aware of the pharmaceutical packages content (triple- blind, randomized clinical trial). However the anesthesiologist and student were aware of the drugs and groups in order to prevent the inadvertent intra and postoperative events.

Patients were kept NPO (nil per os) for 8 hours preoperatively. Both groups received the same standard intravenous drug regimens for hypotensive general anesthesia: 0.3 mg/kg midazolam, 2.5 mg/kg remifentanyl, 1 mg/kg lidocaine, and 100-200 µg/kg/min propofol infusion intravenously were employed to stabilize blood pressure and heart rate in optimal range. Neuromuscular relaxation was achieved with 0.5 mg/kg atracurium, the patients also received 3-4 cc/kg/h Ringer solution in order to achieve proper hydration goal.

As a matter of fact the volatile anesthetics and nitrous oxide were avoided in favor of a total intravenous anesthetic (TIVA) based on propofol and remifentanil infusions titrated by the anesthesia care team. Morphine and codeine were avoided intraoperatively and postoperatively to prevent PONV probability.

All subjects underwent the same surgical technique (bilateral sagittal split osteotomy for mandibular setback, Lefort I osteotomy for maxillary advancement and genioplasty if necessary). Duration of surgery was noted in checklist.

Patients were monitored by pulse oximetry, electrocardiography, thermal probe and capnography, also the heart rate and mean arterial pressure (MAP) were checked carefully during the operation in order to find out any bradycardia or sever hypotension status. To reverse the anesthesia at the end of surgeries, muscle relaxant effect was neutralized by Neostigmine (0.04 mg/kg IV) and Atropine (0.02 mg/kg IV).

Both groups underwent similar antibiotic therapy with intravenous Cefazolin (1gr, 1hour preoperatively and every 6 hours postoperatively). Additionally 8 mg intravenous Dexamethasone was prescribed in both groups (1hour preoperatively, every 4 hours intraoperatively and every 8 hours postoperatively); in order to reduce edema, inflammation and pain.

As it was proved in previous studies that above mentioned drugs were individually stronger than placebo for PONV prevention, therefore the placebo was eliminated in the present study and the authors just compared the efficacy of ondansetron-dexamethasone versus clonidine-dexamethasone.

No blood transfusion were required during the operation. Postoperatively, all patients were transferred to the maxillofacial surgery ward for 24 hours.No oral intake was allowed for patients in the first 6 hours after recovery from anesthesia, so the dextrose-saline serum was administered intravenously.

PONV and pain was assessed and observed by specially trained nursing staff without knowledge treatment groups. (Every 15-30 min in recovery room and later once every hour in the surgical ward for the first postoperative day.)

Post-operative pain was measured subjectively, using a visual analog scale (VAS), in such a way that pain was recorded from 0 (representing no pain) to 10 (representing the most severe pain).The investigator documented the severe pain (VAS≥7) incidence in checklist.

For the first 24 hours after anesthesia, the presence or absence of nausea and vomiting (by simply yes or no) was assessed in both groups. Nausea was defined as the subjectively unpleasant sensation associated with the involuntary attempts to vomit without discharge of stomach contents, whereas vomiting was defined as an actual discharge of gastric contents from the mouth. Any side effects in postoperative period were also recorded.

Metoclopramide (0.2 mg/kg, intravenously) was used as a rescue antiemetic. It was administered when patients vomited or when nausea was intense, with more than 15 min duration. For cases who faced intolerable severe pain (VAS≥7), Apotel (1g, intravenously) was prescribed as a rescue analgesic. The time when these rescue drugs were prescribed was also noted in check list.

The patients discharged in the second day after surgery with stable vital signs. Finally the checklists were encoded and sent for statistical analyses.

In the next stage of data analysis, decoding was performed. Data were analyzed using SPSS for Windows (V. 16, SPSS Inc., Chicago, IL). Qualitative variables were expressed as percentage while quantitative variables stated as mean ± SD (standard deviation). We employed Mann–Whitney, student’s t-test, chi-square and Fisher’s exact test for analysis. *P*-value less than 0.05 were considered statistically significant.

## Results

In the present study, a total of 30 patients including 14 males (46/7%) and 16 females (53/3%) with average age of 23/9±3/9 and age range of 18 to 32 years were investigated during 9 months. All of the cases had class III jaw deformity.

8 females (53.3%) and 7 males (46.7%) were studied in each group (ondansetron or clonidine), therefore the distribution of sex was similar in both. Chi-Square test manifested no statistical difference in sex distribution between clonidine and ondansetron group (*p*=1.00).

Age average in ondansetron group was 23.4±3.8 years and in clonidine group was 24.4±4.0. Therefore, age difference between our study groups was insignificant according to T-test (*p*=0.490).

**Figure 1:**
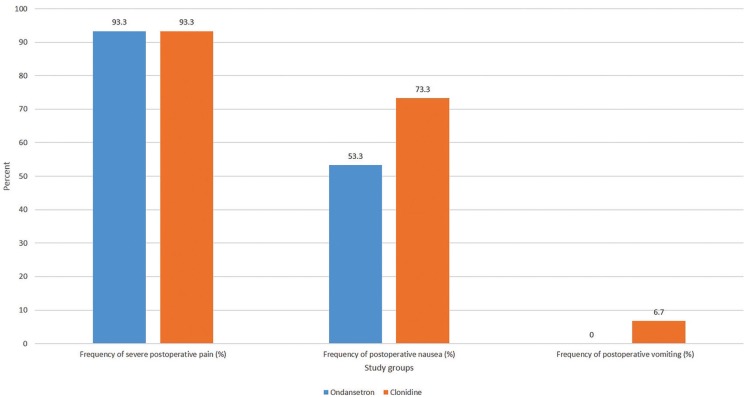
The comparison of postoperative severe pain, nausea and vomiting frequency in ondansetron versus clonidine group.

Diagram 1 illustrates the frequency of post-operative severe pain, postoperative nausea and vomiting in the study groups (Fig. 1).

The frequency of severe post-operative pain (VAS≥7) requiring rescue doses of analgesics in each group was 14 cases (93.3%), which is entirely identical in both groups regarding the ficher’s exact test (*P*=1.00).

The frequency of post-operative nausea (PON) in ondansetron and clonidine groups was 8 (53.3%) and 11 cases (73.3%) respectively. Even though the frequency of PON in the ondansetron group was less than clonidine, but it did not have any significant difference between two groups according to the chi-Square test (*p*=0.256).

Interestingly, the post-operative vomiting (POV) was not observed in ondansetron group (0%), but only 1 patient had POV in clonidine group (6.7%). However, the ficher’s exact test manifested no statistical difference for POV between groups (*p*=1.00).

 Table 1 depicts that there was no significant difference in BMI (Body mass Index) between study groups (*p*=0.903). Although duration of surgery in ondansetron group (166±60.8 min) was a little more than clonidine group (148.7±68.1 min), but they had no significant statistical difference (*p*=0.468).

**Table 1:**
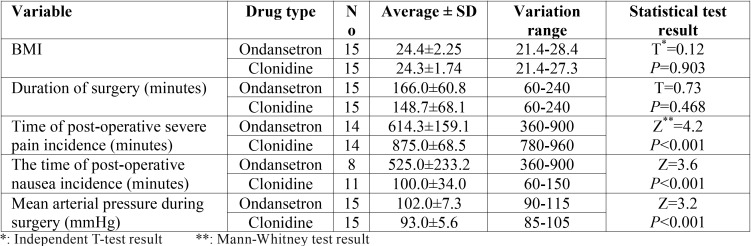
The comparison of BMI, Duration of surgery, Mean arterial pressure during surgery and time of postoperative severe pain and nausea between ondansetron and clonidine group.

Post-operative nausea in ondansetron group occurred significantly later than clonidine group patients, regarding the Mann-Whitney test (525.0±233.2 vs 100.0±34.0 min; *p*<0.001). On the contrary, the incidence time of post-operative severe pain or in other words the analgesia time in clonidine group (875.0±68.5 min) was significantly more than ondansetron one (614.3±159.1 min) according to the Mann-Whitney test (*p*<0.001).

Patients’ mean arterial pressure during surgery in Clonidine group was significantly lower than Ondansetron one (93.0±5.6 vs 102.0±7.3 mmHg; *p*<0.001)  Table 1.

**Figure 2:**
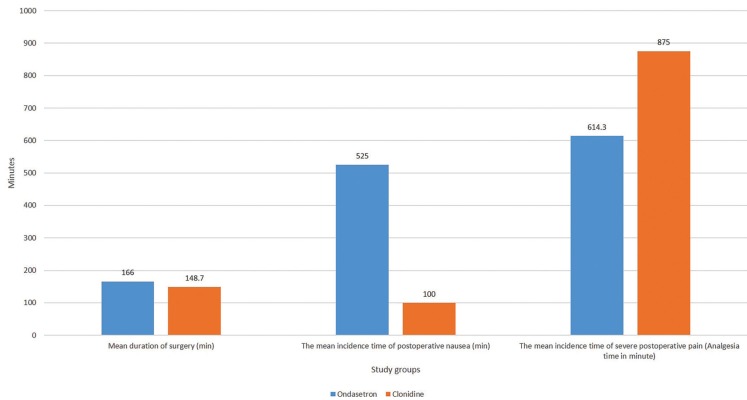
The comparison of mean arterial pressure, mean incidence time of postoperative nausea and severe pain (analgesia time) in ondansetron versus clonidine group.

Diagram 2 illuminates the comparison of the average duration of surgery, the mean incidence time of nausea and severe pain (analgesia time) between ondansetron and clonidine group (Fig. 2).

63.3% of all patients who underwent orthognathic surgeries with more than 3 hours duration showed PON. Furthermore 80% of patients in clonidine group and 46.7% in ondansetron confronted nausea in the case of surgery duration more than 3h. Chi-Square test showed the significant correlation between postoperative nausea incidence and surgery duration longer than 3hours (*P*=0.050).

The incidence of PON in females appeared to be 73.7% and in males 26.3%. In other words, the incidence of postoperative nausea in women was significantly more than men according the chi-Square test (*p*=0.003). However the incidence of severe postoperative pain turned out to be 53.6% in women and 46.4% in men, which had no significant difference (*P*=1.00).

Also by BMI increase in this study, the incidence rate of nausea rose. There was a significant relation between BMI and PON in accordance with the ficher’s exact test analysis (*P*<0.001).

 Table 2 depicts that the surgery type in 20 cases (66.6%) was bimaxillary orthognathic surgery (Bimax), from which 7 cases (23.3% of total) had genioplasty too. The isolated one jaw orthosurgery was done for 10 patients (33.4%), from which 6 (20%) had isolated mandibular osteotomy and 4 cases (13.4%) had maxillary lefort I osteotomy surgeries. In addition, 9 (30%) of all 30 patients required genioplasty ( Table 2).

**Table 2:**
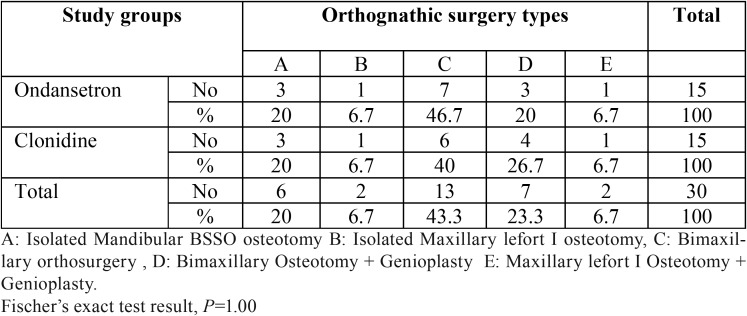
The frequency of orthognathic surgery types in study groups.

Ficher’s exact test analysis revealed both clonidine and ondansetron groups roughly resembled one another in regard to frequency of orthognathic surgery types (*P*=1.00).

PON prevalence in bimax orthosurgery was 68.4% (36.8% alone and 31.6% with genioplasty), also nausea frequency in isolated maxillary lefort I was 21% (10.5% alone and 10.5% with genioplasty) and in isolated mandibular osteotomy was 10.5%. On the other hand, the severe postoperative pain was more frequent in bimax cases (67.9%), followed by isolated mandibular osteotomy (21.4%) and then isolated maxillary lefort I (10.7%) respectively. However these mentioned differences were not statistically significant. (*P*=0.204 and 0.336).

The  Table 3 shows the postoperative pain and nausea prevalence for each surgery type including genioplasty in detail ( Table 3).

**Table 3:**
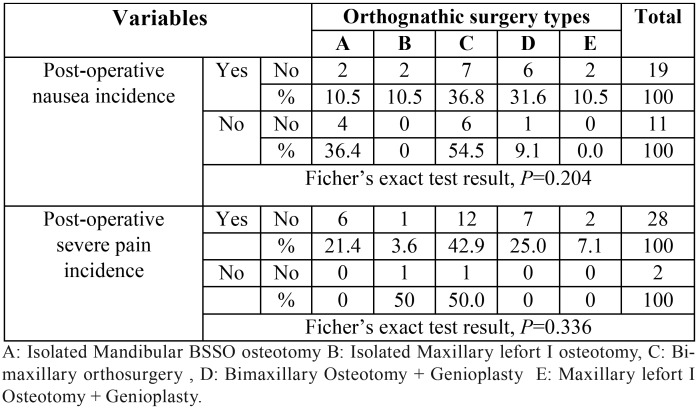
The postoperative pain and nausea prevalence for each orthognathic surgery type.

It should be noted that no major adverse events such as bradycardia or severe hypotension were observed in either group. The patients had no problem in follow ups.

## Discussion

Postoperative nausea and vomiting (PONV) is one of the most undesirable complications related to general anesthesia and appears to be the second common reason for long-term hospitalization (2,3,5-7,11,16).

It complicates the post-anesthesia and postsurgical care and also can result in adverse consequences such as dehydration, esophageal rupture, wound dehiscence, bleeding, hematoma, aspiration of gastric contents and possible death (1-5,8). This issue must receive special attention as PONV causes severe post-operative pain, prolongs post-anesthesia care unit (PACU) stay increases patients’ dissatisfaction and is of high importance due to costs and problems imposing on patients and health care system (1-5,8,11,21,22).

In spite of significant advances in the area of postoperative nausea and vomiting (PONV) and the introduction of new antiemetic agents, the incidence of PONV may be as high as 30-92% in surgical population depending on the type of surgery and patients’ risk factors (2,3).

Although many studies have shown the highest prevalence of PONV among ophthalmologic (80%), ear (70%), intra-abdominal (40 to 70%) and laparoscopic (40 to 77%) surgeries, few reports have focused on this complication after oral and maxillofacial surgeries, especially orthognathic procedures (1,3,9). It should be noted PONV prevention for orthognathic surgical patients remains an under-investigated domain of clinical care (1,2).

The literatures demonstrate a high incidence of both PON and POV after orthognathic operation, even after the last updated consensus guidelines for the management of PONV after general surgery in 2007 (1,2).

Silva *et al.* in 2006 performed the large retrospective review of PONV after orthognathic surgery on 514 patients. They found it had a high prevalence in the first 24 hours comparing to other maxillofacial operations with a particularly high frequency (56%) after bimaxillary osteotomies (3).

Philips *et al.* studied 204 subject after orthognathic surgeries in 2015. They reported the incidence of PON and POV without premedication was 67% and 27% respectively (1).

Unfortunately only limited literatures analyzed the prevalence of PONV and related risk factors as well as the proper management specifically in orthognathic surgery field up to now (1-3,10,23). Hence, further studies are needed to develop effective protocols for preventing this common and unpleasant problem in maxillofacial patients with intermaxillary fixation.

The etiology of PONV is complex and involves a number of interrelated pathways (1,6,7). Regarding the systematic reviews, the risk factors including the younger age, female gender, obesity (BMI more than 30 kg/) and nutritional habits as well as previous history of emesis and motion sickness are the independent predictors for PONV. Moreover, the surgery type and duration (longer than 2-3 hours), the anesthesia drugs particularly volatile agents and opioid analgesics can lead to PONV emergence, especially after orthognathic surgeries (1-3,5-8,9).

In fact, the multifactorial etiology of PONV is better addressed by multimodal approach regarding the recent articles (1-3,5,8,9). Administration of antiemetic drugs to prevent or mange the PONV is an important tool that should be properly used (1-5,8,9). It has been suggested that the use of more than one prophylactic antiemetic drug, acting at different receptor sites is more effective than the use of a single drug especially in high risk patients, however there is no consensus regarding the optimal prophylactic antiemetic regimen nowadays (1-5,8,9).

Regarding to mentioned facts, a multimodal protocol (e.g. ondansetron-dexamethasone or clonidine-dexamethasone) was applied in the present study and also volatile anesthetic was avoided. It is well established that the use of perioperative opioid narcotics would be associated with PONV (1,5,24,25), therefore the effective postoperative pain control would be an important issue in maxillofacial field for patient comfort and safety.

Although it is proven many routine drugs such as metoclopramide and anti-histamines may decrease PONV, but they might cause adverse effects such as xerostomia, hypotension, excess sedation, delusion and extra pyramidal symptoms (15,16,21).

Various researches proved that the prophylactic influence of Ondansetron (5HT3 receptor antagonist) is more effective than Metoclopramide (Dopamine receptor antagonist) on PONV, especially in those who undergo chemotherapy (4,15,24,26). It does not cause the side effects such as Dystonia, bradykinesia and extrapyramidal syndrome(15,16,21). Ondansetron can prevent PONV for 6-12 h after surgery (5).

The recent studies declared ondansetron might attach to µ opioid receptor as an antagonist which leads to analgesic effects especially in neuropathic pains. It is noteworthy that prescribing ondansetron as an analgesic in contrast to opioid narcotics, includes no major adverse effects (15,18-20).

Clonidine is a α2 agonist which reduces blood pressure centrally and peripherally and prevent cardiovascular reactions due to sympathoadernergic response resulting from intubation and laryngoscopy procedures (5,7,10). It also plays sedative and hypotensive roles for anesthesia with less cost and complications compared to benzodiazepines and opioids (5-7,10,11,14).

Khezri *et al.* reported that clonidine provided a better pain relief after cesarean section compared to fentanyl (14).

Since a high sympathetic tone and catecholamine release may trigger nausea and vomiting, a general reduction in sympathetic outflow caused by clonidine could also have contributed to the reduction of PONV (5,7,10).

It has onset of action 0.5-1 h and longer duration of action of up to 12h (5,10).Although the bradycardia and severe hypotension are the clonidine complications (5,10), but no cases in our study confronted them.

Various researches reported the positive impact of clonidine prescription on hemodynamic stability and reducing the incidence of PONV after ear, strabismus and breast cancer surgeries as well as appendectomy and thyroid surgeries (5-7).

Mohammadi and Tabrizi *et al.* stated the advantages of clonidine in their articles. It proposes hemodynamic stability during orthognathic surgery and rhinoplasty by decreasing the bleeding and reducing mean arterial pressure (10).

Dexamethasone is a Glucocorticoid with antiemetic impact especially in chemotherapy patients. It can reduce tissue inflammation and reduce the ascending parasympathetic impulses to the vomiting center. Regarding the literatures, dexamethasone has a good prophylactic effect for PONV when given one hour prior to surgery (4,5,8).

Bano and Fazal wadood *et al.* reported the administration of ondansetron plus dexamethasone is more effective than ondansetron or dexamethasone separately for PONV management (8), thus we applied this combined protocol in our research. In addition, as it was stated before in articles, multifactorial etiology of PONV is better addressed by multimodal approach (1,2,5,12).

To the best of our knowledge, our study was the first triple blind clinical trial which compared the efficiency of clonidine versus ondansetron premedication on postoperative pain and PONV after orthognathic surgeries.

Considering the inclusion and exclusion criteria and after omitting the confounding factors, the current study included 30 class III healthy patients (2 groups of 15 cases). This sample size was identical to Mohammadi *et al.* study which studied the effect of clonidine premedication on hemodynamic status in orthognathic surgeries (10).

The study drugs dose selections for premedication (clonidine, ondanstron and dexamethasone), their prescription protocol and also the rescue antiemetic and analgesic drugs selection (metoclopramide and Apotel) to control PONV and pain were adopted from Shipla, Taheri and Alizadeh *et al.* studies (5-7).

The intravenous Apotel, which is a non-opioid analgesic and does not have increasing effect on nausea and vomiting, was prescribed in cases that confronted post-orthosurgical severe pain in current study, relevant to Shipla *et al.* and Alizadeh *et al.* studies (5,6).

While the PONV occurs primarily within the first 24 hours postoperatively which can lead to significant morbidity (3,5,6), therefore the assessment of pain, nausea and vomiting was performed in the first 24 hours postoperatively in our research, which was in compliance with Taheri, Alizadeh, Bano, Fazal Wadood, Shilpa and Elhakim *et al.* studies (5,6,8). Also the intervals in which we evaluated these variables postoperatively were in harmony with Shipla and Elhakim *et al.* articles (5).

Since previous experiments by Taheri, Elhakim, Alizadeh and Bano *et al.* revealed effectiveness of either clonidine or ondansetron alone, compared with placebo on PONV and pain reduction, therefore we did not utilize placebo in the present study (5-8,10). In fact, regarding the high possibility of POV after orthognathic, we did not include a placebo group for ethical reasons (5). Instead, similar to Shipla *et al.* study on thyroidectomy patients, we compared effects of oral clonidine or ondansetron premedications (both combined with IV dexamethasone) on PONV and pain after orthognathic surgery (5).

Regarding the retrospective study of Philips et al., the incidence of PON and POV without premedication was 67% and 27% respectively after orthognathic surgeries (1).

In our study, postoperative nausea (PON) in ondansetron-dexamethasone group was occurred in 53.3% cases compared to clonidine-dexamethasone in 73.3%, while postoperative vomiting (POV) incidence in ondansetron and clonidine groups was 0% and 6.7% respectively, in this research. Hence, it can be concluded both drugs would decrease POV effectively however ondansetron was more effective in nausea prevention.

Our research findings highlight that the analgesia time in clonidine group was significantly more than ondansetron one (875.0±68.5 min vs 614.3±159.1 min; *p*<0.001). This was very similar to Shipla *et al.* study results after thyroidectomy, as they reported the analgesia time in clonidine and ondansetron groups was 919 vs 642 minutes (1). Regarding to literature, clonidine has a longer duration of action up to 12h (5,6).

Post-operative nausea in ondansetron group occurred significantly later than clonidine group in our study, (525.0±233.2 vs 100.0±34.0 min; *p*<0.001). It was in agreement with Shilpa *et al.* study which demonstrated the incidence time of PON in ondansetron group was within 360-720 min and in clonidine within 60-120 min after thyroidectomy (5). It was similar finding to the present research, as the time range of PON incidence in our study was within 360-900 minutes in ondansetron versus 60-150 postoperatively in clonidine group. Regarding to literatures, ondansetron can prevent PONV for 6-12 h postoperatively (5,15).

Mean arterial pressure (MAP) was averagely 93±5/6 mmHg in Clonidine group and 102±7/3 mm Hg in Ondansetron. However both MAP values were appropriate from anesthetic point of view, but MAP was significantly lower in Clonidine group (*p*<0.001) that corresponds to Mohammadi and Tabrizi *et al.* studies since clonidine leads to hemodynamic stability, hypotension and decreases bleeding during operation (10).

Regarding Apfel, Brooks and Silva *et al.* studies, PONV occurs more frequently in youngeradults which was correspondent to our findings (2,3,9).

Controversies are existed regarding the BMI and PONV relation, as Philips *et al.* believed that BMI≥30 correlated with PONV while some authors like Gan and brooks et al opposed any correlations (1,2,27). However the relation between BMI and nausea was significant in our experiment. (The more BMI, The more PONV incidence.) 

Nausea incidence in females was observed significantly more than males. It is well-known that adult males are less likely to experience PONV than females due to fluctuation in women hormone levels during the menstrual cycle, which matched with Philips, Silva and Shipla *et al.* studies (1,3,5).

The significant relationship was found between postoperative nausea incidence and surgery duration longer than 3hours due to accumulation of emetogenic factors related to general anesthesia.This correlation was similar to Silva and Brooks et al. results which suggested that the obese patients might have a higher prevalence of PONV, especially after long operations (longer than 3 hours) (2,3).

In current study, PON incidence in patients who had Bimaxillary orthoganatic surgery (Bimax) was more than single jaw surgeries. Also Nausea incidence in maxillary osteotomy was observed more than mandibular osteotomy surgery. Sever pain was more frequent in Bimax patients, following by isolated mandibular osteotomy and then maxillary lefort I surgery.

The results were relevant to Philips, Silva and Brooks *et al.* reports (1-3). One explanation of these results may be the greater average length of surgery time and tissue trauma for bimaxillary surgeries versus isolated maxillary or mandibular surgeries.It is emphasized in the literature that the longer the surgery, the higher the incidence of PONV will be (1-3,5,6).

Actually isolated maxillary surgery showed a higher prevalence of PONV than did mandibular procedures alone, despite the fact that the length of the surgery was similar in both groups. It can be explained by greater postoperative bleeding, blood swallowing probability and the hypotensive anesthesia which is often used during maxillary osteotomies (1-3,10).

In addition, genioplasty as a complementary operation works as an additional and increasing factor for duration of surgery and bleeding risk which will result in higher PONV incidence (1-3).

Mandibular osteotomy puts more pressure on musculoskeletal components of temporomandibular joint, therefore the pulled muscles cause higher post-operative pain (1-3,10).

Also, other treatment options such as 5HT3 antagonists, such as granisetron, palonosetron, and ramosetron, are used for PONV prophylaxis (28,29).

Limitations and suggestions

One of the present study limitations was the sample size, so we suggest recruiting a larger sample size with higher number of orthognathic surgery patients for future studies to signify the correlation between other factors. Another limitation was the fact that this study was a single center research so low number of surgeons participated. Future studies will work best if they gain more maxillofacial surgeons’ attentions and compare diverse treatment centers.

In addition, PONV and pain were only monitored during the first 24 hours postoperatively in present research. This could be better if patients’ check-ups continued more, albeit Silva *et al.* determined that PONV would occur most commonly within the first 24 hours postoperatively in orthognathic patients (3).

Despite the limitations, the outcome of this study was quite satisfying since it proposed an effective multimodal protocol for prevention of PONV and post-operative severe pain after orthognathic surgeries

## Conclusions

Our study showed that ondansetron with dexamethasone premedication was more effective in controlling PONV after orthognathic surgery compared to clonidine with dexamethasone group. However, the analgesia time was longer with clonidine than ondansetron group, but on the contrary there is no significant difference between the ondansetron and clonidine group in terms of postoperative severe pain occurrence.

Therefore premedication with oral ondansetron accompanied by intravenous dexamethasone could be proposed as an effective multimodal protocol to reduce both PONV and post-operative severe pain in orthognathic surgeries without major and significant side effects.
